# Trends in organ donation in England, Scotland and Wales in the context of the COVID-19 pandemic and ‘opt-out’ legislation

**DOI:** 10.1371/journal.pone.0306541

**Published:** 2024-07-31

**Authors:** Stephen O’Neill, Karen Thomas, Leah McLaughlin, Paul Boadu, Lorraine Williams, Mustafa Al-Haboubi, Jennifer Bostock, Jane Noyes, Nicholas Mays

**Affiliations:** 1 Department of Health Services Research and Policy, Policy Innovation and Evaluation Research Unit, London School of Hygiene and Tropical Medicine, London, United Kingdom; 2 Clinical Research and Development, Royal Marsden NHS Foundation Trust, London, United Kingdom; 3 School of Medical and Health Sciences, Bangor University, Bangor, United Kingdom; Australian Red Cross Lifeblood, AUSTRALIA

## Abstract

**Background:**

In May 2020, England implemented soft ‘opt-out’ or ‘deemed consent’ for deceased donation with the intention of raising consent rates. However, this coincided with the COVID-19 pandemic, making it difficult to assess the early impact of the law change. Wales and Scotland changed their organ donation legislation to implement soft opt-out systems in 2015 and 2021 respectively. This study provides a descriptive analysis of changes in consent and transplant rates for deceased organ donation in England, Scotland and Wales.

**Methods:**

Logistic regression and descriptive trend analysis were employed to assess the probability of a patient who died in critical care becoming a donor, and to report consent rates using data, respectively, from the Intensive Care National Audit and Research Centre (ICNARC) in England from 1 April 2014 to 30 September 2021, and from the Potential Donor Audit for England, Scotland and Wales from April 2010 to June 2023.

**Results:**

The number of eligible donors in April-June 2020 were 56.5%, 59.3% and 57.6% lower in England, Scotland and Wales relative to April-June 2019 (pre-pandemic). By April-June 2023, the number of eligible donors had recovered to 87.4%, 64.2% and 110.3%, respectively, of their levels in 2019. The consent rate in England, Scotland and Wales reduced from 68.3%, 63.0% and 63.6% in April-June 2019 to 63.2%, 60.5% and 56.3% in April-June 2023.

**Conclusions:**

While the UK organ donation system shows signs of recovery from the COVID-19 pandemic, the number of eligible potential donors and consent rates remain below their pre-pandemic levels.

## 1 Introduction

When an organ fails or can no longer function properly, a transplant may be the only viable option for survival. While organ donation can save or improve the lives of people with organ failure, there is a shortage of organs available for transplantation. There are currently around 7,000 people on the UK Transplant waiting list, and over 420 people died while waiting for a transplant in 2021 [1]. Increasing organ donation rates can allow more patients to receive life-saving transplants and lead to better allocation of resources for transplant programs, more efficient use of hospital resources and improved patient outcomes [2]. In the UK, 39% of organ donors are living donors, with living donor transplantation representing 21% of total transplant activity [3]. The organ donation system is thus heavily reliant on organ donations from the deceased, which include both donors after brain death (DBD) and donors after circulatory death (DCD). However, a relatively small number of people die in circumstances optimal for organ donation. This, in conjunction with historically relatively low rates of opt-in to deceased organ donation, meant that in the UK, as in many countries, there was a gap between the number required and availability of organs for donation, motivating efforts to increase the pool of organ donors.

### 1.1 Organ donation legislation in the UK

Although in practice the circumstances leading to deceased organ donation has always been a complex interface of acute bereavement, what the deceased may have expressed in life, family dynamics, circumstances of the death, and health system capacity, as an overall principle before 2015 the UK was based on an ‘opt-in’ system, requiring individuals explicitly to choose to allow their organs to be donated after death. Since 2015, different parts of the UK have been moving at different pace towards systems of organ donation consent based on the concept of ‘deemed consent’. In principle, this presumes that individuals are ‘deemed’ willing to donate their organs unless they explicitly choose to ‘opt out’. The British Medical Association (BMA) has consistently argued that a move from a default opt-in system to deemed consent, as part of a package of supportive changes, would greatly improve donation rates [4, 5]. An international systematic review of studies published from 2006 to 2016 reported that opt-out systems tended to increase deceased donation and transplantation rates [6]. It was thus suggested that moving from an opt-in to an opt-out system could save 700 lives a year in England [7], although contrary views have been expressed by some clinicians and researchers [5, 8–11].

Following the Human Transplantation (Wales) Act 2013, Wales moved to a ‘soft’ opt-out system of consent for organ donation. The law change was fully enacted on 1^st^ December 2015 [12, 13] and was preceded by a 2 year media campaign ran by the Welsh Government and National Health Service Blood and Transplant to inform people about the change, which was seen as vital to the Act’s success [14].

On 20^th^ May 2020, the Organ Donation (Deemed Consent) Act 2019 came into effect in England, introducing a similar soft opt-out system of presumed consent. In principle, all adults in England are now considered to have agreed to be an organ donor when they die unless they have expressed a decision not to donate (either on the Organ Donor Register or telling someone) or are in an excluded group, such as being aged under the age of 18, or not having lived in England for more than 12 months. Families of potential donors are consulted before any donation takes place, and are given the opportunity to provide information about the potential donor’s views on organ donation. The ‘soft’ opt-out means that the family can still override the deceased decision whether they registered, discussed it, or decided to do nothing and therefore were presumed to support organ donation, and their consent could be deemed under the new legislation.

More recently, the Human Tissue (Authorisation) (Scotland) Act 2019 was passed by the Scottish Parliament, coming into effect on 26^th^ March 2021. The legislation for Scotland is ‘deemed authorisation’, meaning if an individual has not confirmed whether they want to be a donor or not, they will be considered to be willing to donate their organs and tissue when they die. Northern Ireland also moved to an opt-out system on 1^st^ June 2023. Below, we focus on England, Scotland and Wales. This sequence of similar law changes in the three countries at different times offers a natural experiment with the potential to assess the impact of deemed consent for the first time in a wider UK context.

### 1.2 The COVID-19 pandemic

Unfortunately, analysing the impact of the move to deemed consent in England and Scotland following the law change in Wales is challenging as the effects were conflated with the effects of the COVID-19 pandemic. The COVID-19 pandemic significantly affected healthcare systems worldwide, leading to concerns about the availability of donor organs and the ability to perform transplant surgery. Aubert et al [15] report that, across 22 countries, the overall number of kidney, liver, lung, and heart transplants from human donors fell by 16% during the first wave of COVID-19. Ugur and Pérez [16] considered data from IRODaT (International Registry in Organ Donation and Transplantation) between 2010 and 2021 for 32 countries in Europe, and found that the COVID-19 pandemic substantially reduced organ donation, deceased and especially live kidney transplantation rates. Figure A1 in the S1 Appendix shows the daily number of Covid-19 cases over time for context.

Manara et al [17] report a reduction in trauma and other emergency department admissions in the UK of over 50% during the first lockdown (23^rd^ March to 10^th^ May 2020). However, despite the pandemic, deceased donation (transplant) activity was reportedly sustained at 75% of normal levels in 2020/21 [18]. Plummer et al [19] used national audit data from NHS Blood and Transplant (NHSBT) to compare the first 12 months after the pandemic (11^th^ March 2020 and 10^th^ March 2021) with the corresponding 12 months immediately pre-pandemic. They report that during the first wave (11^th^ March to 1^st^ September 2020) of the pandemic, referrals to NHSBT of potential organ donors were inversely related to the number of people with COVID-19 undergoing mechanical ventilation in intensive care. However, in the second wave (2^nd^ September 2020 to 10^th^ March 2021), this was reversed, with a positive relationship observed. Overall, there were fewer eligible donors and a lower total number of donations when compared with the pre-pandemic period, but the proportion of eligible donors who proceeded to donation (27%) was unchanged.

The introduction of deemed consent aimed to increase the number of organs available for transplant by increasing the consent rates within the potential donor pool, providing an associated increase in the number of organs available for transplantation and in donation rates. However, increases in the consent rate may not necessarily lead to increases in the transplant rate, since a deceased person who has consented to organ donation during their lifetime does not always proceed to donation as some families override the deceased decision, some DCD donors do not die in a suitable timeframe to allow donation to occur, and some donors may not have any organs deemed suitable for transplantation. The conversion of potential donors to successful donation tends to be low–a recent study suggested that less than 20% of patients identified as eligible donors went on to donate successfully [20].

### 1.3 Existing evidence

A number of studies have analysed the short term effects of the earliest UK introduction of deemed consent, that in Wales. An early evaluation of the effects of the Welsh legislation change reported that while consent rates had increased in Wales after the law change, they had also increased similarly in the rest of the UK, so the Welsh increase could not be attributed specifically to the legislation change, while the number of deceased donors remained largely unchanged [12]. Albertsen [21] compared Wales with the UK as a whole using a difference-in-differences approach, and reported that concerns expressed by sceptics that deemed consent might even decrease both living and deceased donation rates had not materialised. More recently, Madden et al [13], using a longer follow up period (33 months), concluded that consent rates in Wales had been positively impacted. For England, very early reports indicated that the law change was associated with little change to the consent rates, or organ donations numbers, albeit changes may take longer to manifest or be confounded by the effects of the pandemic [22].

Here we do not seek to disentangle the causal effect of the law changes and the pandemic, but rather conduct a descriptive analysis of changes in consent rates and transplant rates over time in England, Scotland and Wales, focussing on deceased donor consent rates before and after the law changes in each country, and before and after the initial wave of COVID-19.

## 2 Methods

### 2.1 Critical care—ICNARC data analysis

Data from ICNARC on admissions to critical care of patients aged between 20 and 80 years to NHS units in England between 1 April 2014 and 30 September 2021 were used. We reported the total number of reported admissions, deaths, and deaths leading to any organ or tissue donation, by quarterly time periods. Patient characteristics of age group, sex, ethnicity (collected using standard NHS categories and grouped as White; Mixed; Asian; Black; Other; Not stated) and primary reason for admission to critical care (grouped as trauma, cardiovascular, gastrointestinal, neurological, genito-urinary, endothermic, metabolic, thermoregulation and poisoning, haematological or immunological, other) were reported as counts and percentages, for all deaths and by donation status. A logistic model was fitted to estimate the probability of a patient who died in critical care becoming an organ or tissue donor, adjusting for variables previously identified as affecting this decision (age, sex, ethnicity, reason for admission), and for date of admission (grouped into quarters). The adjusted odds ratio for each quarter (with 95% CI) compared to the starting period of April-June 2014 was calculated., to determine whether changing percentages of organ donations were driven solely by changes in the characteristics of patients dying in critical care, or by other factors not measured in these data. Each variable in the logistic model was separately tested for an interaction with quarter, to determine whether or not the association between that variable and the probability of donation remained constant over time, despite the pandemic and law change.

### 2.2 Potential Donor Audit (PDA)

The PDA is a continuous national audit of all patients aged ≤ 80 years who die within an intensive care or emergency department in a UK hospital. For this analysis, we obtained access to anonymised data from NHSBT captured in the PDA for England, Scotland and Wales. Potential donors are defined as those deceased patients who could be solid organ donors. The PDA data include data on both DCD and DBD donors. For DBD, deceased donors’ intensive care treatments are continued after death is confirmed to preserve organs until they can be retrieved, whereas for DCD, organ donation takes place following the diagnosis of death using circulatory criteria. In the UK, the average number of transplantable organs retrieved from DCD donors (2.8) is similar to that from DBD donors (3.2), while DCD donors represented 44% of all deceased organ donors in 2021/22 [23].

The PDA reflects the pool of potential deceased donors whose families may be contacted about organ donation. Both potential DBD donors and DCD donors may be excluded before being approached due to a number of absolute contraindications (ACI) which clinically preclude organ donation as per NHSBT criteria [24]. In addition, potential DCD donors without absolute contraindications may also be excluded due to the DCD screening process before families are approached. Thus, these potential donors will not be captured in the NHSBT consent data. We exclude live donors and limit analysis to individuals aged 20 to 80 years, as in the ICNARC analysis.

We use simple trend analysis to describe variation over time in the rate of consent, defined as the percentage of eligible donor families approached for organ donation discussion where consent/authorisation for donation was ascertained, and the transplant rate, defined as the number of transplants divided by the number consented donors from whom at least one organ was transplanted. Outcomes are defined on a quarterly basis to reduce variability in the measurement, given the relatively low number of eligible donors in Wales and Scotland. While the pandemic is an important confounder preventing reliable estimation of the causal effects of the move towards deemed consent, especially in England and Scotland–to the extent that changes coincide across the nations, we can infer these are more plaubily attributed to the pandemic than to the similar law changes, and conversely where changes are nation-specific, we may infer that these are potentially attributable to the law changes unless countries adapted their organ donation system differently in face of the pandemic which is unlikely since NHSBT operates similarly across the UK.

Data from the PDA were available for 94,598 patients referred to the organ donation service for consideration of organ donation over 53 quarters of data running from Q2 2010 (i.e. April-June 2010) to Q2 2023 (April-June 2023). Of these, 65,411 were deemed to be eligible DBD or DCD donors, with 61,142 aged between 20 and 80. Unless otherwise stated, analysis was limited to the 36,038 (England = 31,576; Scotland = 2,633; Wales = 1,829) eligible potential donors aged between 20 and 80 years where the family was approached for discussion of organ donation. Consent was provided for 22,634 (England = 19,863; Scotland = 1,629; Wales = 1,132) of these potential donors.

Data were accessed on 14th October 2022 (ICNARC) and 12^th^ July 2023 (PDA) and did not contain information that could identify individuals.

### 2.3 Ethics approval

Ethics approval for the study was obtained as part of a larger Evaluation of the Organ Donation (Deemed Consent) Act, 2019 England from the London School of Hygiene and Tropical Medicine ethics committee (Ref: 26427) and HRA (Ref: 21/NW/0151). The need for consent for the analysis of routine administrative data was waived by the ethics committee.

## 3 Results

### 3.1 Critical care—ICNARC data analysis

**Fig 1:**
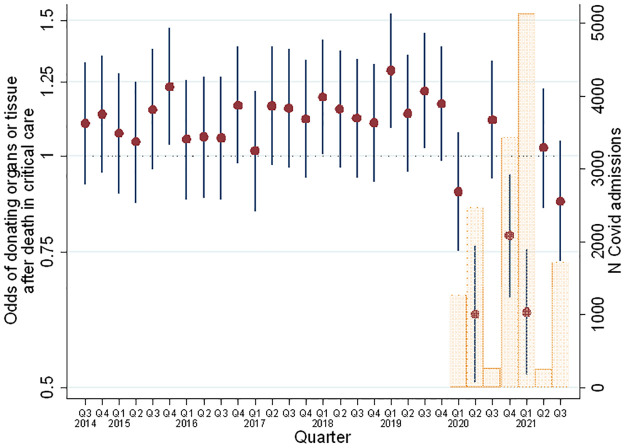
Adjusted odds ratio (with number of Covid-19 admission) of donating organ tissue after death in critical care over time.

Overall, organ or tissue donations made up 6.4% of all deaths in adult critical care recorded over the time period, and 0.8% of all admissions, or 7.1% if we consider only the pre-COVID-19 period (prior to Q1 2020) (Table 1). Donation rates, defined as the proportion of deaths leading to organ and/or tissue donation, varied over time but with an overall rising trend between 2014 and 2019 (also seen in the PDA analysis, below), which was then followed by major fluctuations during the pandemic. This pattern remained after adjusting for patient characteristics (Table 2) known to affect donation rates, suggesting these changes were driven by something other than changes in the recorded patient characteristics (Fig 1). The influence of recorded patient characteristics on likelihood of donation remained consistent over time for ethnicity and sex, but changed for age and presence of significant medical history, likely reflecting changes in eligibility during the pandemic rather than in decisions made by next of kin (Table 3).

**Table 1:** Admission, deaths, and donation rates by time period based on ICNARC data.

Quarter	Admissions		Deaths			Organ and/or tissue donation	
	All (N)	Covid (N)	All (N)	% of admissions	Covid (N)	N	% of deaths
2014 q2	33,208	0	4,174	12.6	0	278	6.7
2014 q3	34,955	0	4,397	12.6	0	309	7
2014 q4	37,073	0	4,988	13.5	0	352	7.1
2015 q1	35,547	0	4,923	13.8	0	326	6.6
2015 q2	37,269	0	4,483	12	0	309	6.9
2015 q3	37,728	0	4,337	11.5	0	317	7.3
2015 q4	38,860	0	4,961	12.8	0	362	7.3
2016 q1	39,301	0	5,474	13.9	0	321	5.9
2016 q2	39,596	0	4,752	12	0	308	6.5
2016 q3	40,756	0	4,515	11.1	0	297	6.6
2016 q4	41,788	0	5,154	12.3	0	363	7
2017 q1	41,181	0	5,068	12.3	0	313	6.2
2017 q2	41,638	0	4,707	11.3	0	348	7.4
2017 q3	41,707	0	4,691	11.2	0	339	7.2
2017 q4	43,734	0	5,324	12.2	0	358	6.7
2018 q1	43,593	0	5,738	13.2	0	405	7.1
2018 q2	44,811	0	4,936	11	0	362	7.3
2018 q3	44,622	0	4,661	10.4	0	350	7.5
2018 q4	45,112	0	4,952	11	0	366	7.4
2019 q1	44,173	0	5,250	11.9	0	413	7.9
2019 q2	45,403	0	4,784	10.5	0	361	7.5
2019 q3	46,100	1	4,923	10.7	0	393	8
2019 q4	45,853	1	5,284	11.5	0	399	7.6
2020 q1	43,181	3,322	6,103	14.1	1,270	318	5.2
2020 q2	33,586	7,846	5,597	16.7	2,476	184	3.3
2020 q3	40,489	965	4,546	11.2	259	361	7.9
2020 q4	43,451	10,593	7,235	16.7	3,427	282	3.9
2021 q1	42,596	16,080	8,494	19.9	5,120	242	2.8
2021 q2	41,337	1,213	4,666	11.3	254	325	7
2021 q3	40,552	6,457	5,771	14.2	1,716	301	5.2

**Table 2:** Characteristics of deaths in critical care units, by organ donation status based on ICNARC data.

	Donors	Non-donors	All deaths
	(N = 9,962)	(N = 144,917)	(N = 154,879)
Male, n (%)	5734 (57.6)	88543 (61.1)	94277 (60.9)
Age, median (IQR)	57 (46, 67)	66 (55, 73)	65 (55, 73)
Age, n (%)			
<50	3186 (32.0)	22937 (15.8)	26123 (16.9)
50–59	2489 (25.0)	26299 (18.1)	28788 (18.6)
60–69	2412 (24.2)	41523 (28.7)	43935 (28.4)
70–79	1811 (18.2)	50134 (34.6)	51945 (33.5)
80+	64 (0.6)	4024 (2.8)	4088 (2.6)
Ethnicity, n (%)			
White	8840 (88.7)	120185 (82.9)	129025 (83.3)
Mixed	49 (0.5)	890 (0.6)	939 (0.6)
Asian	235 (2.4)	9686 (6.7)	9921 (6.4)
Black	111 (1.1)	4149 (2.9)	4260 (2.8)
Other	206 (2.1)	3230 (2.2)	3436 (2.2)
Not stated	521 (5.2)	6777 (4.7)	7298 (4.7)
Any history of severe conditions, n (%)	628 (6.3)	32880 (22.7)	33508 (21.6)
Primary system affected/reason for admission			
Trauma	1450 (14.6)	7765 (5.4)	9215 (6.0)
Cardiovascular	1965 (19.7)	35242 (24.3)	37207 (24.0)
Gastrointestinal	272 (2.7)	20445 (14.1)	20717 (13.4)
Neurological (including eyes)	5108 (51.3)	16847 (11.6)	21955 (14.2)
Genito-urinary	57 (0.6)	6960 (4.8)	7017 (4.5)
Endocrine, Metabolic, Thermoregulation and Poisoning	125 (1.3)	3644 (2.5)	3769 (2.4)
Haematological/Immunological	20 (0.2)	3074 (2.1)	3094 (2.0)
Other	16 (0.2)	2281 (1.6)	2297 (1.5)

**Table 3:** Multivariable logistic model for probability of a death in critical care resulting in donation based on ICNARC data.

Variable		Odds Ratio (95% CI)		p-value from test of interaction with quarter
Age (in years)		0.97	(0.97, 0.97)	0.0001
Male sex		0.88	(0.84, 0.92)	0.1868
Ethnic group:	White	1		0.3287
	Mixed	0.59	(0.43, 0.8)	
	Asian	0.31	(0.27, 0.35)	
	Black	0.27	(0.22, 0.33)	
	Other	0.64	(0.55, 0.74)	
	Not stated	0.86	(0.78, 0.95)	
Any significant medical history		0.32	(0.3, 0.35)	0.0163
Primary system affected/reason for admission:				n/a*
	Cardiovascular	2.35	(2.17, 2.55)	
	Gastrointestinal	0.62	(0.54, 0.71)	
	Neurological (including eyes)	11.15	(10.36, 12)	
	Genito-urinary	0.40	(0.31, 0.53)	
	Endocrine, Metabolic, Thermoregulation and P.	1.17	(0.96, 1.41)	
	Haematological/Immunological	0.38	(0.24, 0.59)	
	Trauma	6.59	(6.04, 7.2)	
	Other	0.28	(0.17, 0.46)	
Quarter:	2014 q2	1		
	2014 q3	1.10	(0.92, 1.32)	
	2014 q4	1.13	(0.95, 1.35)	
	2015 q1	1.07	(0.9, 1.28)	
	2015 q2	1.04	(0.87, 1.25)	
	2015 q3	1.15	(0.96, 1.38)	
	2015 q4	1.23	(1.03, 1.47)	
	2016 q1	1.05	(0.88, 1.26)	
	2016 q2	1.06	(0.88, 1.27)	
	2016 q3	1.06	(0.88, 1.27)	
	2016 q4	1.16	(0.98, 1.39)	
	2017 q1	1.02	(0.85, 1.21)	
	2017 q2	1.16	(0.97, 1.39)	
	2017 q3	1.15	(0.97, 1.38)	
	2017 q4	1.12	(0.94, 1.33)	
	2018 q1	1.19	(1.01, 1.42)	
	2018 q2	1.15	(0.97, 1.37)	
	2018 q3	1.12	(0.94, 1.34)	
	2018 q4	1.10	(0.93, 1.31)	
	2019 q1	1.29	(1.09, 1.53)	
	2019 q2	1.14	(0.95, 1.35)	
	2019 q3	1.22	(1.02, 1.44)	
	2019 q4	1.17	(0.99, 1.39)	
	2020 q1	0.90	(0.75, 1.07)	
	2020 q2	0.62	(0.51, 0.76)	
	2020 q3	1.11	(0.94, 1.33)	
	2020 q4	0.79	(0.66, 0.95)	
	2021 q1	0.63	(0.52, 0.76)	
	2021 q2	1.02	(0.86, 1.22)	
	2021 q3	0.87	(0.73, 1.05)	

*Not calculated because of small sample sizes within admission groups

In the COVID-19 era (post-2020 Q1) death rates tended to be higher than in the pre-COVID-19 era, while conversely the number and proportion of deaths that led to organ and/or tissue donation, tended to be lower. Of the deaths in critical care units, those that became donors were more likely to be male, less than 60 years of age, White, be admitted for trauma or neurological (including eyes) reasons and less likely to have a history of severe conditions (Tables 2 and 3).

### 3.2 Potential Donor Audit

**Fig 2:**
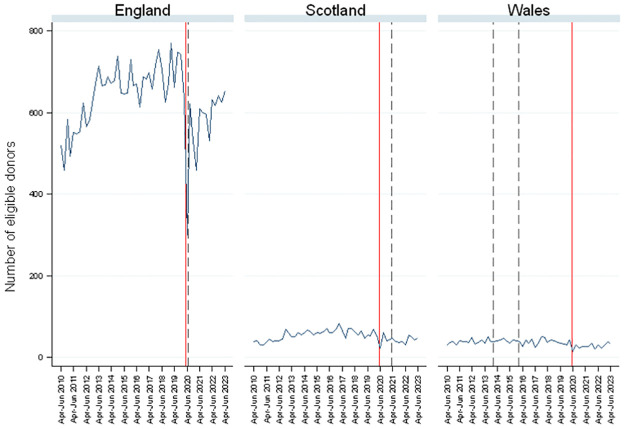
Number of eligible donors by country and quarter.

Fig 2 shows that most eligible donors are residents of England which is unsurprising given the disparity in population sizes. Hence, other reported values (e.g. consent rates) for Scotland and Wales tend to be more variable than for England. The number of eligible donors was increasing in England from 2010 to 2013, but was fairly stable thereafter. There was a large drop in the number of eligible donors during the first wave of COVID-19. Given the population size, the absolute drop was smaller in Scotland and Wales. However, in percentage terms relative to 12 months previously (i.e. preceding the pandemic), the changes in the number of eligible donors in Q2 of 2020 were more similar: 56.5% lower in England compared to 59.3% and 57.6% lower for Scotland and Wales, respectively. By the last period included in the analysis (April-June 2023), the number of eligible donors had recovered to 87.4%, 64.2% and 110.3% of their levels pre-pandemic in England, Scotland and Wales, respectively, albeit the absolute numbers were small (<50) in the latter two countries meaning that percentage changes should be interpreted with great care. NHS Blood and Transplant (NHSBT) commenced screening for Severe Acute Respiratory Syndrome Coronavirus 2 (SARS-CoV-2) in deceased organ donors on 19 March 2020, and also revised its organ donation acceptance criteria, prioritising DBD over DCD and younger donors [19]. As shown in Table A1 in S1 Appendix, this resulted in a change in the age composition of consented donors (See Table 1 in Plummer at al [19] for details on age acceptance criteria).

**Fig 3:**
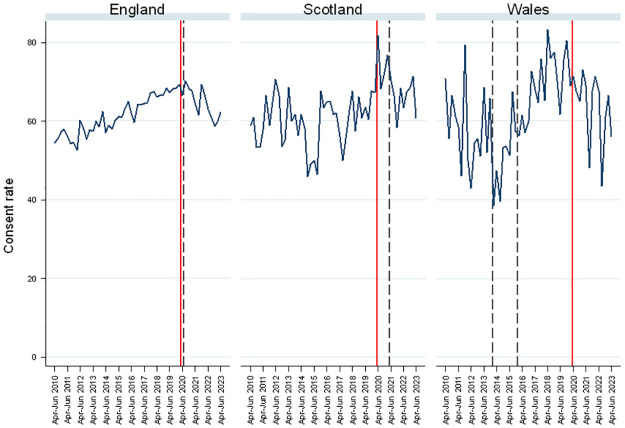
Consent rate by country and quarter.

The consent rate broadly tracks the number of eligible donors, with a large drop in the peak pandemic period followed by a recovery (not shown). However, while the consent rate dropped in the COVID-19 era (Fig 3), the drop is not as stark as for the number of eligible donors. The consent rate in Scotland has returned close to the level pre-COVID-19, while this has not occurred in England or Wales (Fig 3). The dashed lines in Fig 3 correspond to the introduction of the respective ‘deemed consent’ Acts. For Wales, the leftmost dashed line indicates the media campaign run by the Welsh Government and NHSBT for two years preceding full implementation on 1 December 2015.

In England, the consent rate had been increasing over the decade before the pandemic and the law change (Fig 3; left panel). While consent rates dropped following the introduction of the Act, since similar drops were also seen in Scotland (Fig 3; middle panel) and Wales (Fig 3; right panel). Thus, we attribute this drop to the pandemic, rather than the Act. In Scotland and Wales, consent rates are volatile due to the lower number of eligible donors (driven by population size). In Scotland, following its legislation, there has been a drop in consent rates, but there are similar patterns in the other countries so this cannot be attributed to the Scottish law change. In Wales, following the initial period of the media campaign in 2013/14, there was a decrease in consent rates [leftmost dashed line]. However, this cannot be directly attributable to the law change since it preceded it or the media campaign since it also coincided with adverse media coverage of a particular adverse event where two recipients died from a kidney donor who was infected with meningitis, the first ever case in the UK. There was an increase in consent rates following the Act’s implementation [rightmost dashed line], but there was also an increase over this period in England and Scotland, so, again, this cannot be confidently attributed to the legislative change in Wales.

Table A2 in S1 Appendix reports the consent rate by age group for each nation, and overall. For England, consent rates are broadly similar by age group, while more variation is observed for Wales and Scotland, given the smaller number of eligible potential donors aged between 20 and 80 years where the family was approached for discussion of organ donation. Table A3 in S1 Appendix reports the number of eligible potential donors approached, and the consent rate for DBD and DCD potential donors by financial year. We see a large (32%) reduction in the number of eligible potential donors approached in 2020, coinciding with the start of the pandemic. The reduction was greater in percentage terms for DCD (-43%) than for DBD (-17%). Table A4 in S1 Appendix reports the number of potential donors and consent rates by quarter, along with the percentage change relative to the corresponding quarters in 2019, i.e. before COVID-19. A large drop in April-June 2020 coincided with the first wave of the pandemic, with the drop particularly large for DCD (74.1%). By the final quarter for which data were available (April—June 2023), the percentage had recovered to be 4.7% above the corresponding quarter in 2019, while DBD remained 14.1% below its level in 2019. While there was a large drop in the number of potential donors, the consent rate tended to reduce less noticeably. Towards the end of 2022, consent rates remained 6.5 and 1.7 percentage points below their 2019 levels for DCD and DBD, respectively.

Figure A2 in S1 Appendix shows the number of consented donors from whom at least one organ was transplanted over time. This follows a broadly similar pattern to the number of consented donors, dropping sharply in the pandemic period in England and not yet having fully recovered, although this pattern is less evident in the other nations. This again suggests that a return to 2019 levels of activity has not yet occurred. Figure A3 in S1 Appendix shows the transplant rate and provided some evidence that a greater number of organs have been transplanted per consented donor in the COVID-19 era.

To assess the change in patterns of consent associated with the law changes more directly, Figure A4 in S1 Appendix graphs the percentage of eligible donors in four consent groupings defined by whether the patient (a) had expressed an opt in decision, (b) had expressed an opt out decision, (c) met deemed criteria specific to each nation, or (d) had expressed no decision or the deemed criteria were not met. For each country, the proportion of donors in the last group dropped following the law change in that country. As one might expect, the proportion in the deemed consent group increased rapidly from zero after the law changes. However, in all three countries, this remained below 50% of the potential donors included in the analysis. In England, the proportion that had opted in was increasing before the law change but this has not continued after the law change which may be due to the law in theory providing a default of opt-in without the individual having to take any action.

## 4 Discussion

The findings of this study suggest that organ and tissue consent and donation rates have changed significantly over time, with an increase between 2014 and 2019 for the three countries combined, albeit this is dominated by changes in England. However, this trend was interrupted by major fluctuations during the COVID-19 pandemic. While the UK organ donation system shows signs of recovery from the pandemic, donation rates, the number of eligible potential donors and consent rates remain below their pre-pandemic levels. These findings are more likely to be attributable to the pandemic rather than the switch to deemed consent, given the trends observable in Wales which had moved to deemed consent long before the pandemic. The fact that consent rates in England had been increasing steadily before COVID-19 and the law change calls into question, to some degree, the assumed need for the change to deemed consent, especially since this was accompanied by warnings from experts that the change would be unlikely to be beneficial and could harm the organ donation system [25–28]. During the pandemic, death rates were higher, while the number and proportion of deaths leading to organ and tissue donation and transplantation were lower. The number of eligible donors dropped sharply during the first wave of COVID-19, with a slow recovery in England. The number of consented donors from whom at least one organ is transplanted remains below pre-pandemic levels.

Despite having an opt-in system, rates of DCD donors in the US have increased steadily since the mid-1990′s implying that presumed consent is not a necessary condition for increasing consent rates [29]. Spain has often been acknowledged as achieving the highest rate of organ donation from deceased donors. While this is sometimes attributed to its legislative environment, it is important to note that other factors are at work that may explain why Spain performs better than other countries such as the UK [25]. The UK has relatively low provision of intensive care facilities [30], while Spain tends to use older donors [31] and provides indirect financial incentives for hospitals to take an active role in organ donation [32]. Spain also contributes towards the funeral expenses of deceased donors [33, 34] while there are also religious differences between the countries which may also play a role [33]. Presumed consent has, therefore, been described as ‘a distraction’ in understanding what drives higher rates of organ donation in Spain [35]. Among the lessons that can be learned from Spain [36] are the importance of continuous governmental commitment to, and support for, the programme, the absence of financial barriers and the appropriate training for professionals [36]. Policies that nurture a culture of trust and confidence in the organ donation and transplantation program are also likely to be important. Ultimately, overall success in increasing consent rates requires that the public trusts the system of deceased organ donation.

Although over the long-term consent rates in deceased organ donation have been steadily increasing, year-on-year this figure remains highly volatile. Unexplained dips have on the whole been attributed to various national scandals within the NHS such as the Liverpool care pathway and the Alder Hey organs scandal, as well as more specific cases. While unrelated directly to deceased organ donation, most agree there is an inverse relationship between trust and a system which has been largely built upon the principle of altruism [32]. Given the extraordinary context in which opt-out was implemented (a pandemic), and particular events which followed in England (the murder of George Floyd, vaccine hesitancy, the death of the Queen, the Ukraine War, a cost of living crisis and an NHS consistently depicted in the media as at or beyond breaking point) it is perhaps unsurprising that the consent rates have not yet realised a sustained increase. This study is part of a wider study designed to look more closely at some of these more nuanced factors [37] and to help explain further why (as yet) deceased organ donation consent rates appear to continue to remain one of the challenges to increasing the number of organs available for transplant in the UK system. Overall, the findings suggest that a return to pre-pandemic levels in terms of number of eligible potential donors, consent rates and donation rates has not yet occurred, though the organ donation system shows signs of recovery from the COVID-19 pandemic. These results provide important insights into the lasting impact of the COVID-19 pandemic on organ and tissue donation rates and highlight the need for continued efforts to increase donation rates irrespective of whether the system is based on opt-out or opt-in principles.

## Supporting information

S1 Appendix (DOCX)
