# Supplementary material for: Trends in organ donation in England, Scotland and Wales in the context of the COVID-19 pandemic and ‘opt-out’ legislation
Source: PLoS One. 2024 Jul 31;19(7):e0306541. doi: 10.1371/journal.pone.0306541 (PMC11290620; doi:10.1371/journal.pone.0306541)
Supplement: S1 Appendix — (DOCX) [file pone.0306541.s001.docx]

**Appendix:**

**Table A1: Proportion of eligible potential donors whose family were approached and for whom consent was given within each age group**

| **Age**  **group** | **January**  **to**  **March**  **2020** | **April**  **to**  **June**  **2020** | **July**  **to**  **September**  **2020** | **October**  **to**  **December**  **2020** |
| --- | --- | --- | --- | --- |
|  |  |  |  |  |
| **Less than 20** | 5.3% | 5.3% | 5.4% | 5.4% |
| **20 to 25** | 4.1% | 3.5% | 2.2% | 2.7% |
| **25 to 30** | 3.4% | 9.7% | 4.4% | 2.2% |
| **30 to 35** | 4.7% | 6.2% | 6.6% | 4.2% |
| **35 to 40** | 5.1% | 8.4% | 5.0% | 5.4% |
| **40 to 45** | 6.7% | 8.8% | 5.8% | 6.1% |
| **45 to 50** | 8.7% | 11.9% | 10.0% | 7.1% |
| **50 to 55** | 10.1% | 18.5% | 12.6% | 12.7% |
| **55 to 60** | 11.4% | 11.0% | 15.6% | 14.5% |
| **60 to 65** | 12.6% | 7.9% | 12.0% | 12.0% |
| **65 to 70** | 11.2% | 4.8% | 9.4% | 11.8% |
| **70 to 75** | 9.1% | 4.0% | 8.4% | 10.5% |
| **75 to 80** | 7.1% | 0.0% | 2.4% | 5.1% |
| **80 to 85** | 0.4% | 0.0% | 0.2% | 0.2% |
|  |  |  |  |  |
| **All ages** | 100% | 100% | 100% | 100% |

**Table A2: Consent rate by age group for each nation:**

| Age | England  (N=31,576) | Scotland  (N=2,633) | Wales  (N=1,829) | Total  (N=36,038) |
| --- | --- | --- | --- | --- |
|  | % | % | % | % |
| 20 to 25 | 64.3 | 62.2 | 67.3 | 64.3 |
| 25 to 30 | 65.8 | 68.7 | 69.0 | 66.2 |
| 30 to 35 | 61.5 | 64.1 | 68.8 | 62.1 |
| 35 to 40 | 64.6 | 70.8 | 67.5 | 65.2 |
| 40 to 45 | 63.1 | 68.6 | 65.4 | 63.6 |
| 45 to 50 | 63.7 | 65.6 | 68.2 | 64.0 |
| 50 to 55 | 63.9 | 61.4 | 61.4 | 63.6 |
| 55 to 60 | 62.6 | 60.3 | 57.8 | 62.1 |
| 60 to 65 | 61.3 | 59.7 | 60.9 | 61.2 |
| 65 to 70 | 62.8 | 60.2 | 59.6 | 62.4 |
| 70 to 75 | 61.7 | 54.1 | 58.6 | 61.1 |
| 75 to 80 | 63.0 | 49.4 | 56.6 | 62.0 |
|  |  |  |  |  |
| Total | 62.9 | 61.9 | 61.9 | 62.8 |

**Table A3: Number of eligible potential donors approached, and the consent rate for DBD and DCD potential donors by financial year**

| Financial  Year (April to March) | Number of eligible potential donors approached | | |  | Consent rate | | |
| --- | --- | --- | --- | --- | --- | --- | --- |
|  | DCD | DBD | All |  | DCD | DBD | All |
|  |  |  |  |  | % | % | % |
| 2010 | 1,281 | 914 | 2,195 |  | 52.0 | 65.3 | 57.5 |
| 2011 | 1,492 | 958 | 2,450 |  | 50.2 | 62.9 | 55.2 |
| 2012 | 1,679 | 987 | 2,666 |  | 52.5 | 68.4 | 58.4 |
| 2013 | 1,835 | 1,113 | 2,948 |  | 54.8 | 68.2 | 59.9 |
| 2014 | 1,848 | 1,136 | 2,984 |  | 52.8 | 67.1 | 58.2 |
| 2015 | 1,774 | 1,154 | 2,928 |  | 58.5 | 69.6 | 62.8 |
| 2016 | 1,702 | 1,207 | 2,909 |  | 59.6 | 69.0 | 63.5 |
| 2017 | 1,719 | 1,339 | 3,058 |  | 61.1 | 72.7 | 66.2 |
| 2018 | 1,640 | 1,364 | 3,004 |  | 63.0 | 72.5 | 67.3 |
| 2019 | 1,679 | 1,323 | 3,002 |  | 65.5 | 72.6 | 68.6 |
| 2020 | 952 | 1,094 | 2,046 |  | 64.6 | 74.1 | 69.7 |
| 2021 | 1,341 | 1,132 | 2,473 |  | 63.2 | 68.9 | 65.8 |
| 2022 | 1,988 | 1,382 | 3,370 |  | 57.6 | 69.0 | 62.2 |
|  |  |  |  |  |  |  |  |
| Total | 20,935 | 15,103 | 36,038 |  | 57.9 | 69.5 | 62.8 |

**Table A4: Number of eligible potential donors approached, and the consent rate for DBD and DCD potential donors by quarter and change from the corresponding quarter in 2019.**

| **Quarter** | **Number of eligible potential donors approached** | | | **% change relative to corresponding quarter in 2019** | | |  | **Consent Rate** | | | **Absolute percentage point change relative to corresponding quarter in 2019** | | |
| --- | --- | --- | --- | --- | --- | --- | --- | --- | --- | --- | --- | --- | --- |
|  |  |  |  |  |  |  |  |  |  |  |  |  |  |
|  | **DCD** | **DBD** | **All** | **DCD** | **DBD** | **All** |  | **DCD** | **DBD** | **All** | **DCD** | **DBD** | **All** |
|  |  |  |  | % | % | % |  | % | % | % | % | % | % |
| 2019: January - March | 462 | 358 | 820 |  |  |  |  | 65.8 | 72.1 | 68.5 |  |  |  |
| 2019: April - June | 402 | 319 | 721 |  |  |  |  | 64.2 | 72.1 | 67.7 |  |  |  |
| 2019: July - September | 458 | 336 | 794 |  |  |  |  | 66.4 | 70.2 | 68.0 |  |  |  |
| 2019: October - December | 439 | 366 | 805 |  |  |  |  | 64.7 | 73.8 | 68.8 |  |  |  |
| 2020: January – March | 380 | 302 | 682 | -17.7 | -15.6 | -16.8 |  | 66.6 | 74.5 | 70.1 | 0.8 | 2.4 | 1.6 |
| 2020: April – June | 104 | 208 | 312 | -74.1 | -34.8 | -56.7 |  | 56.7 | 75.0 | 68.9 | -7.5 | 2.9 | 1.2 |
| 2020: July – September | 334 | 340 | 674 | -27.1 | 1.2 | -15.1 |  | 65.3 | 74.7 | 70.0 | -1.1 | 4.5 | 2.0 |
| 2020: October – December | 253 | 303 | 556 | -42.4 | -17.2 | -30.9 |  | 66.4 | 71.6 | 69.2 | 1.7 | -2.2 | 0.4 |
| 2021: January – March | 261 | 243 | 504 | -43.5 | -32.1 | -38.5 |  | 65.1 | 75.7 | 70.2 | -0.7 | 3.6 | 1.7 |
| 2021: April – June | 344 | 301 | 645 | -14.4 | -5.6 | -10.5 |  | 62.7 | 68.8 | 65.5 | -1.5 | -3.3 | -2.2 |
| 2021: July – September | 332 | 303 | 635 | -27.5 | -9.8 | -20.0 |  | 58.4 | 65.0 | 61.6 | -8.0 | -5.2 | -6.4 |
| 2021: October - December | 354 | 283 | 637 | -19.4 | -22.7 | -20.9 |  | 63.8 | 76.0 | 69.2 | -0.9 | 2.2 | 0.4 |
| 2022: January – March | 311 | 245 | 556 | -32.7 | -31.6 | -32.2 |  | 68.2 | 65.7 | 67.1 | 2.4 | -6.4 | -1.4 |
| 2022: April – June | 358 | 297 | 655 | -10.9 | -6.9 | -9.2 |  | 59.8 | 69.7 | 64.3 | -4.4 | -2.4 | -3.4 |
| 2022: July - September | 379 | 275 | 654 | -17.2 | -18.2 | -17.6 |  | 59.9 | 65.8 | 62.4 | -6.5 | -4.4 | -5.6 |
| 2022: October - December | 380 | 286 | 666 | -13.4 | -21.9 | -17.3 |  | 54.5 | 69.6 | 61.0 | -10.2 | -4.2 | -7.8 |
| 2023: January – March | 430 | 249 | 679 | -6.9 | -30.5 | -17.2 |  | 57.2 | 68.7 | 61.4 | -8.6 | -3.4 | -7.1 |
| 2023: April – June | 421 | 274 | 695 | 4.7 | -14.1 | -3.6 |  | 57.7 | 70.4 | 62.7 | -6.5 | -1.7 | -5.0 |


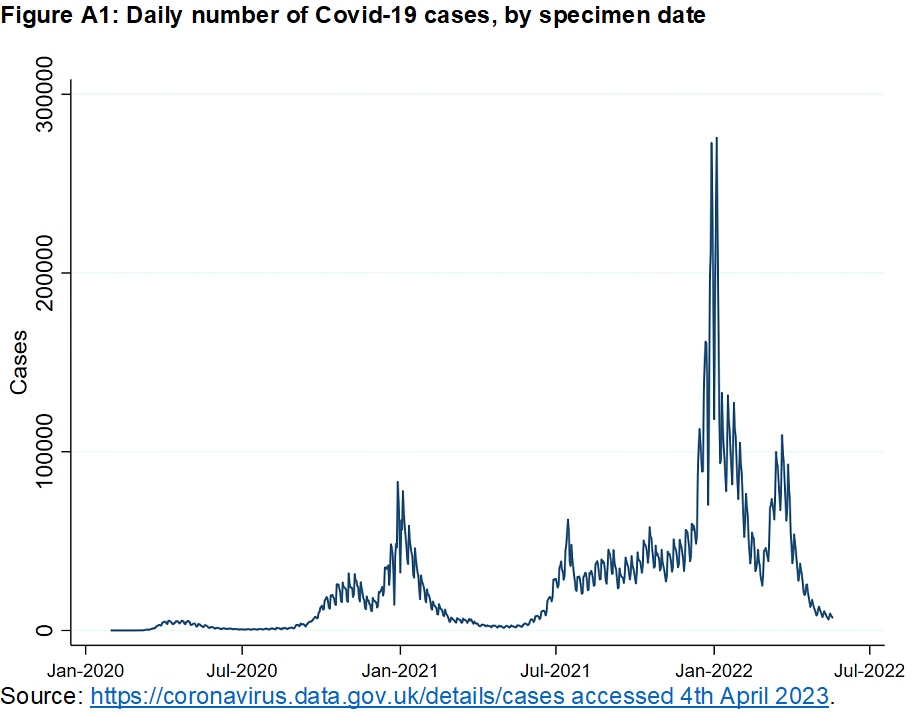


**Figure A1: Daily number of Covid-19 cases, by specimen date**


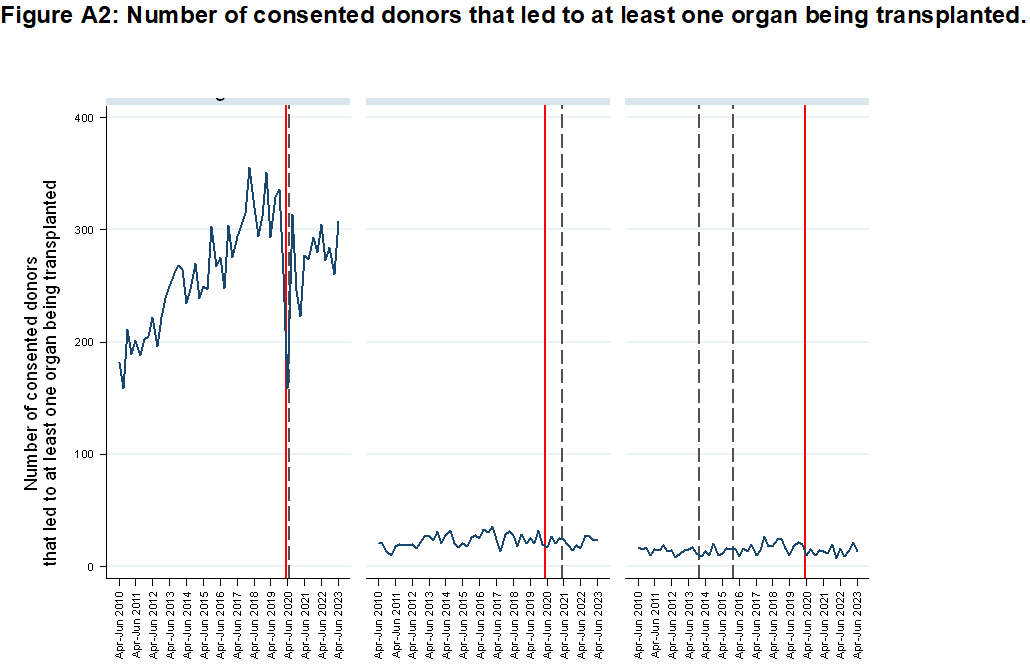


**Figure A2: Number of consented donors that led to at least one organ being transplanted**


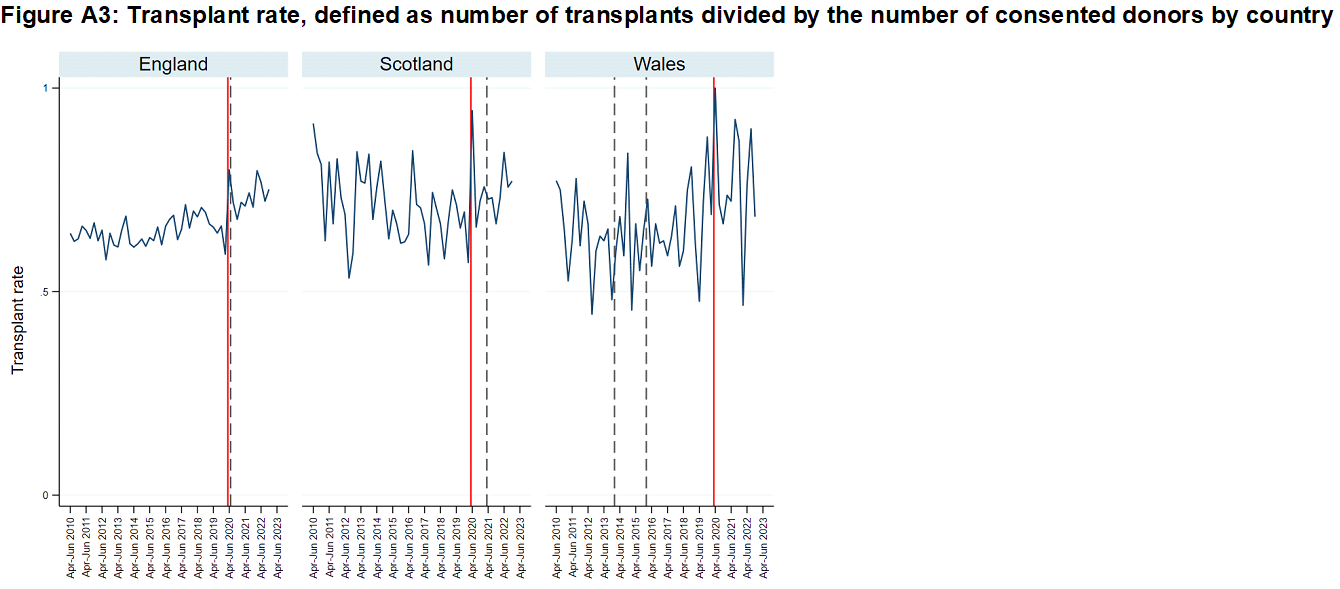


**Figure A3: Transplant rate, defined as the number of transplants divided by the number of consented donors by country**


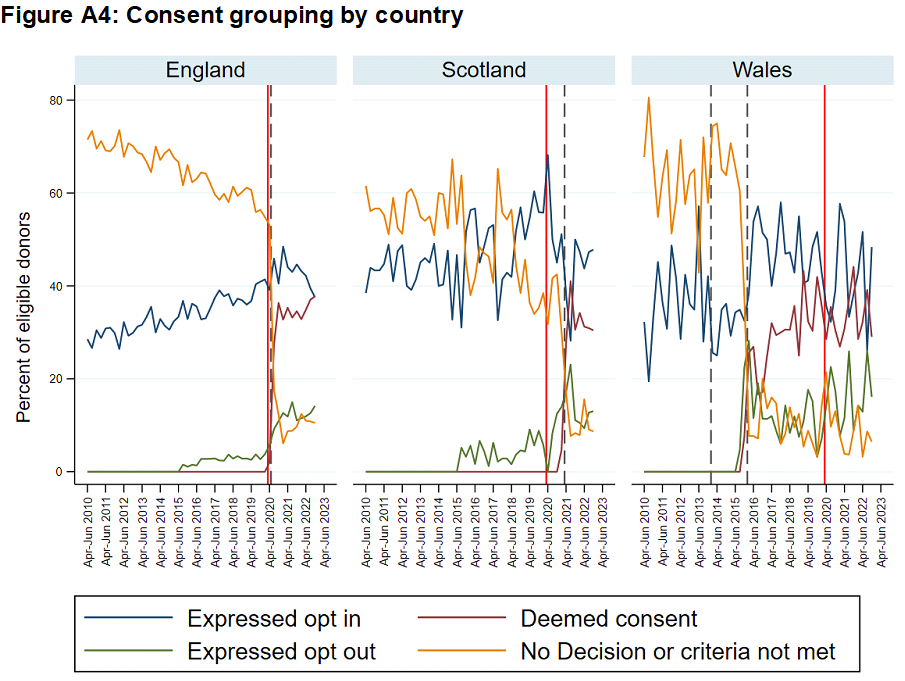


**Figure A4: Consent grouping by country**
